# Tumor-tropic endothelial colony forming cells (ECFCs) loaded with near-infrared sensitive Au nanoparticles: A “cellular stove” approach to the photoablation of melanoma

**DOI:** 10.18632/oncotarget.9511

**Published:** 2016-05-20

**Authors:** Giancarlo Margheri, Angela Zoppi, Roberto Olmi, Silvana Trigari, Rita Traversi, Mirko Severi, Daniele Bani, Francesca Bianchini, Eugenio Torre, Francesca Margheri, Anastasia Chillà, Alessio Biagioni, Lido Calorini, Anna Laurenzana, Gabriella Fibbi, Mario Del Rosso

**Affiliations:** ^1^ Institute for Complex Systems, National Research Council, Sesto Fiorentino, Italy; ^2^ Department of Physics “Enrico Fermi”, University of Pisa, Italy; ^3^ Present address: Plasmatech, Department of Physics “Enrico Fermi”, University of Pisa, Pisa, Italy; ^4^ Institute of Applied Physics “Nello Carrara”, National Research Council, Sesto Fiorentino, Italy; ^5^ Department of Chemistry “Ugo Schiff”, University of Florence, Sesto Fiorentino, Italy; ^6^ Department of Clinical and Experimental Medicine, University of Florence, Florence, Italy; ^7^ Department of Experimental and Clinical Biomedical Science, University of Florence, Florence, Italy; ^8^ Excellence Center for Research, Transfer and High Education ‘Study at Molecular and Clinical Level of Chronic, Inflammatory, Degenerative and Neoplastic Disorders for the Development on Novel Therapies’, Florence, Italy

**Keywords:** gold nanoparticles, endothelial colony forming cells (ECFCs), chemokine (C-X-C motif) receptor 4 (CXCR4), melanoma, tumor thermoablation

## Abstract

In the photothermal treatments (PTs) of tumor, the localization of a high number of near-infrared (NIR) absorbing gold nanoparticles in the tumor mass is still a challenging issue. Here, we propose a promising strategy to deliver therapeutic chitosan-coated gold nanoparticles to tumor cells as hidden cargo of Endothelial Colony Forming Cells (ECFCs) endowed with an innate tumor-tropism. Remarkably, ECFC gold enrichement doesn't affect cell viability and preserves the endothelial lineage characteristics such as capillary morphogenesis and cell migration. We demonstrate that heavily Au-doped ECFCs are able to efficiently warm up the tumor environment, and kill the cancer cells *via* hyperthermic heating both *in vitro* as well as *in vivo*. Thus, we show an excellent thermotransductive property of gold enriched ECFCs and their capability to kill melanoma cells at moderate NIR light intensities.

## INTRODUCTION

Cell-based therapies, namely treatments in which stem or progenitor cells are induced to home within damaged or cancer tissues, and nanomaterial-mediated photothermal ablation using near-infrared (NIR) light have significantly advanced and are poised to become a major pillar of modern medicine.

In the last decade, cell-mediated delivery has been widely used in the therapy of many kinds of tumors, including glioma, head and neck carcinoma and breast cancer [1, 2]. This method appears to be suitable for all kinds of tumor [1], but in reality not all cells can be used to specifically deliver therapeutic payloads. For instance, the shuttle cells should have a high loading capacity for the drugs or nanomaterials and innate tumor tropism [3, 4].

Gold nanoparticles (GNPs) are promising therapeutic tools for the treatment of cancer due to their non-cytotoxic nature, reliable synthesis and functionalization and, most importantly, their tunable plasmonic properties. This allows tailoring gold nanostructures to produce local heating when exposed to near-infrared (NIR) light [5–9]. Although multifunctional nanoparticles have been used as photothermal agents in cancer treatment, at the current stage of development the majority of the administered nanoparticles end up in healthy organs and tissues, even with the assistance of active targeting [10–12]. Moreover particles with longer circulation times, and hence greater ability to target the site of interest, are more exposed to clearance by macrophages [13]. Furthermore, virtually all molecular or nanoparticle-based therapies are inaccessible to the hypoxic areas of tumors that lack blood flow, thus limiting the treatment efficacy [14]. Moreover, it has been reported the effect of PEGylated GNP on deformability and oxygen-delivering ability of the primary functions of erythrocytes [15]. Direct intratumoral injection can be used to circumvent the inefficiency and off-target deposition [16, 17], however the injected nanoparticles generally remain at the site of injection and are unable to penetrate the tumor mass leading to incomplete ablation and disease recurrence.

In the light of these considerations, the current approach to improve delivery of nanodrugs is based on exploiting live cells with natural tumor-homing ability, such as T-cells, monocytes/macrophages and mesenchymal stem cells (MSCs) [18–20].

In this work, we propose alternative NIR-sensitive, tumor tropic cellular vectors, Endothelial Colony Forming Cells (ECFCs), enriched with chitosan-coated Au nanoparticles. ECFCs cells are a sub-class of Endothelial Progenitor Cells (EPC), that, thanks to their excellent tumor-tropism, have been elicited as ideal candidates for cell-based anti-cancer treatments [21]. Recently, this conclusion was supported by direct observation that intravenously injected, ^111^In-oxine labeled ECFCs homed selectively in human melanomas, transplanted in nude mice, by exploiting the CXCR4/SDF1 axis [22]. Alongside, these cells can be readily procured from autologous donors, are easily expanded and modified *ex vivo* [23], limiting possible issues of intolerance or hypersensitivity. Moreover, considering their unique, strategic position at the interface between plasma and interstitial fluid, endothelial cells and their progenitors are endowed with the cellular machinery to perform transcytosis, which implies transporting plasma molecules to the subjacent cells and tissues *in vivo*, whereas *in vitro* the transcytotic cargo vesicles are retained within the cells preventing exocytosis of their content. Finally, being natural “moving gears” *per se* these cells with tumor-tropic features are able to deliver their hidden cargo, which consists of chitosan-coated Au nanoparticles, to the tumor. Chitosan, a highly biocompatible polysaccharide, gives to the nanoparticles an overall positive external electric charge that, other than stabilizing the colloidal solution, promotes two beneficial effects: 1) as the cell membranes have an external negative charge, an improved enrichment is expected as a consequence of the stronger electrically-induced nanoparticle-cell interaction [24]; 2) upon massive loading, chitosan-capped nanoparticles are able to bypass the rapid lysosomal exocitic pathway and remain in the cytoplasm more than 10 days after the enrichment stage. We found that ECFCs exhibit a remarkable avidity of these Au nanoparticles, but at the same time the phenotypical properties of ECFCs are substantially unchanged. We simulated the laser treatment *in vitro* by irradiating a liquid mixture of human A375 melanoma cells and enriched ECFCs, a model that realistically renders the volumetric heat diffusion and the subsequent heat absorption by nearby cells. We found, *in vitro* a massive tumor cells death upon laser irradiation at moderate intensity. Nonetheless, *in vivo* photothermal experiment of gold-enriched ECFCs directly perfused in a tumor mass, showed a considerable necrotization of tumor tissue.

Melanoma is a worldwide increasing pathology, whose incidence in Caucasian population is increasing 3% on a yearly basis. In spite of recent advances in melanoma treatment the patient prognosis is still very poor because of its high multidrug resistance, easy to relapse and low survival rate. With the development of nanotechnology, the use of nano-objects is widely expected to change the landscape of melanoma therapy in the near future.

## RESULTS

### Nanoparticles characterization and ECFC enrichment

**Figure 1:**
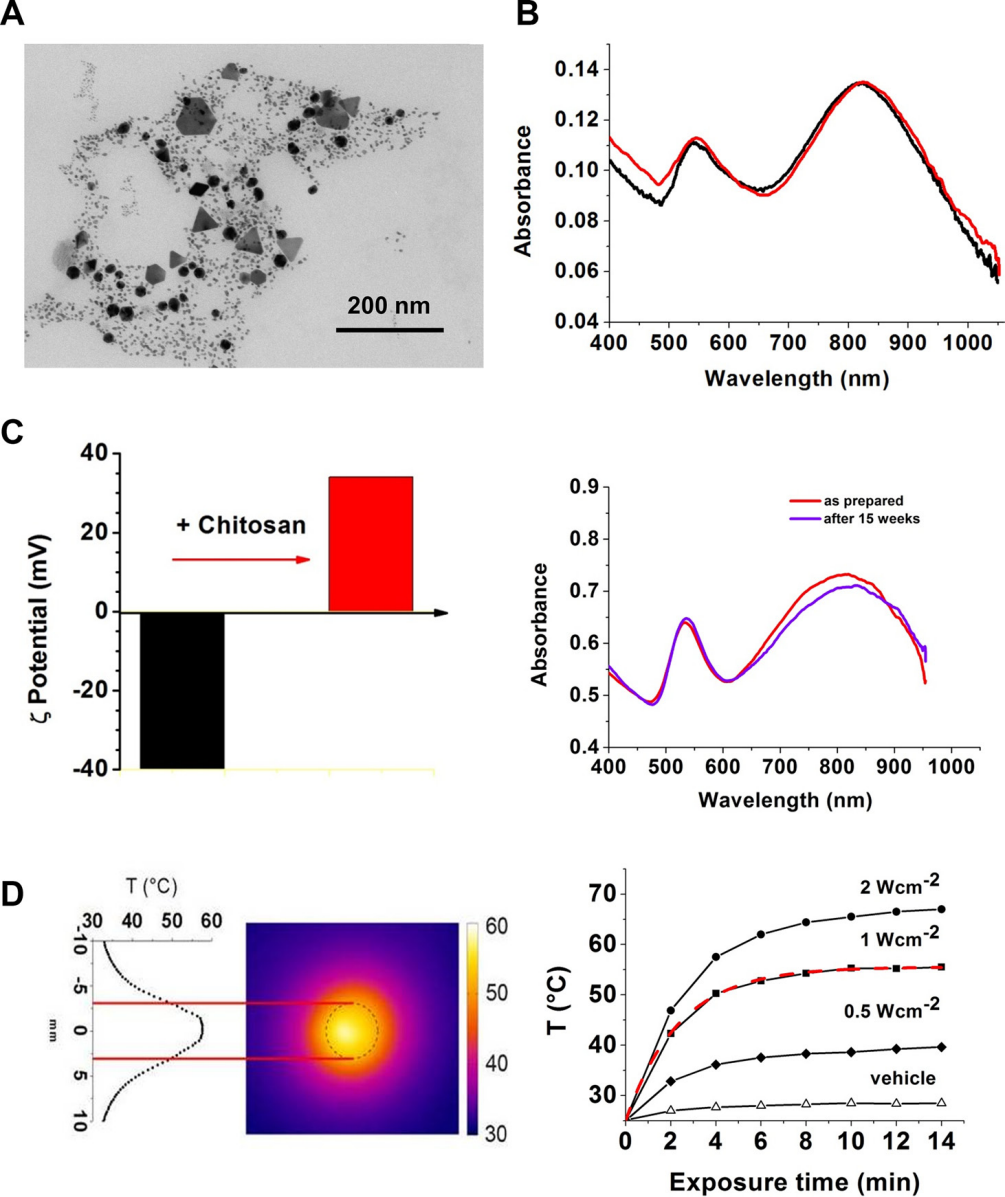
Nanoparticle characterization (**A**) TEM image of Aumix, (**B**) spectrum of as-synthesized Aumix before (black) and after (red) chitosan capping, (**C**) From the left to the right: z potential of nanoparticles before and after chitosan addition. Spectrum of as-prepared chitosan capped Aumix (red) and after 15 weeks (purple) (**D**) From the left to the right: thermography of the NIR-sensitive colloids during irradiation; the dashed circle in the thermography corresponds to the well containing the irradiated sample. Kinetics of temperature increases at three different light intensities. The straight lines are only a guide for the eye. The red line superimposed to the kinetic recorded at 1 W cm^−2^ is the best fit curve calculated to obtain the molar heating rate (MHR,) of the colloid (see text). The calculated initial growing rate is 0.2°C s^−1^.

GNPs were synthesized (~1 hour) by one-step reaction without the assistance of additional templates, capping agents or seed assembly. The product (Aumix) consists of nanoparticles with different shape and size, including small spherical colloid gold particles (diameter < 5 nm) and aspherical gold crystals [25]. Figure 1A shows a typical TEM image of as-produced Aumix. A typical UV-Vis extinction spectrum of the colloidal solution is reported in Figure 1B, with two absorption peaks due to the surface plasmon resonance. The peak centered at around 520 nm is mainly due to the characteristic plasmonic fingerprint of the spherical colloidal nanoparticles and the second component is at NIR band, which is attributed to the dipolar SPR absorption from the anisotropic GNPs [25, 26]. The NIR-band could in principle be enhanced by removing the spherical particles [24, 25], but the modest heating enhancement achievable, later on described, doesn't seem to justify the adoption of the more complex production route.

The surface modification obtained after chitosan addition was confirmed by a red-shift of the absorbance spectrum (Figure 1B) and a change of the colloidal ζ-potential (Figure 1C), that shifted from −40 mV for the as-prepared colloidal dispersion to +32 mV. Even though the as-produced colloidal solutions remained stable *per se* for a variable period of time (from a few days to a week), the addition of chitosan on Aumix (from now on ChAumix) guaranteed a longer-term stability, assessed by recording the plasmonic spectrum immediately after the chitosan addition and after 15 weeks in standard environmental conditions (Figure 1C). Photothermal properties were evaluated by measuring the temperature increase upon irradiation at three light intensities (Figure 1D). A temperature increase of 17.3°C after 120 s was recorded at the intensity of 1 W cm^−2^, not far from that, 19°C, reported by Madsen [19] and Zhang [24] for a colloid synthesized as ours but subsequently refined depriving it of the spherical GNPs and irradiated with the same light intensity.

Alternatively, the photothermal figure of merit of a colloidal solution can be suitably described by the molar heating rate (MHR) [27] which is defined as the temperature growth rate divided by the molar concentration of Au. In our case, the best fitting of the temperature kinetic recorded at 1 Wcm^−2^ NIR intensity (Figure 1D), gives an initial growth rate of 0.2°C s^−1^. Dividing this rate by the Au molar concentration of 1.4 × 10^−3^ M, a MHR of 143°C s^−1^ M^−1^ is derived.

### ECFC vs. A375: Au enrichment and photothermal properties

**Figure 2:**
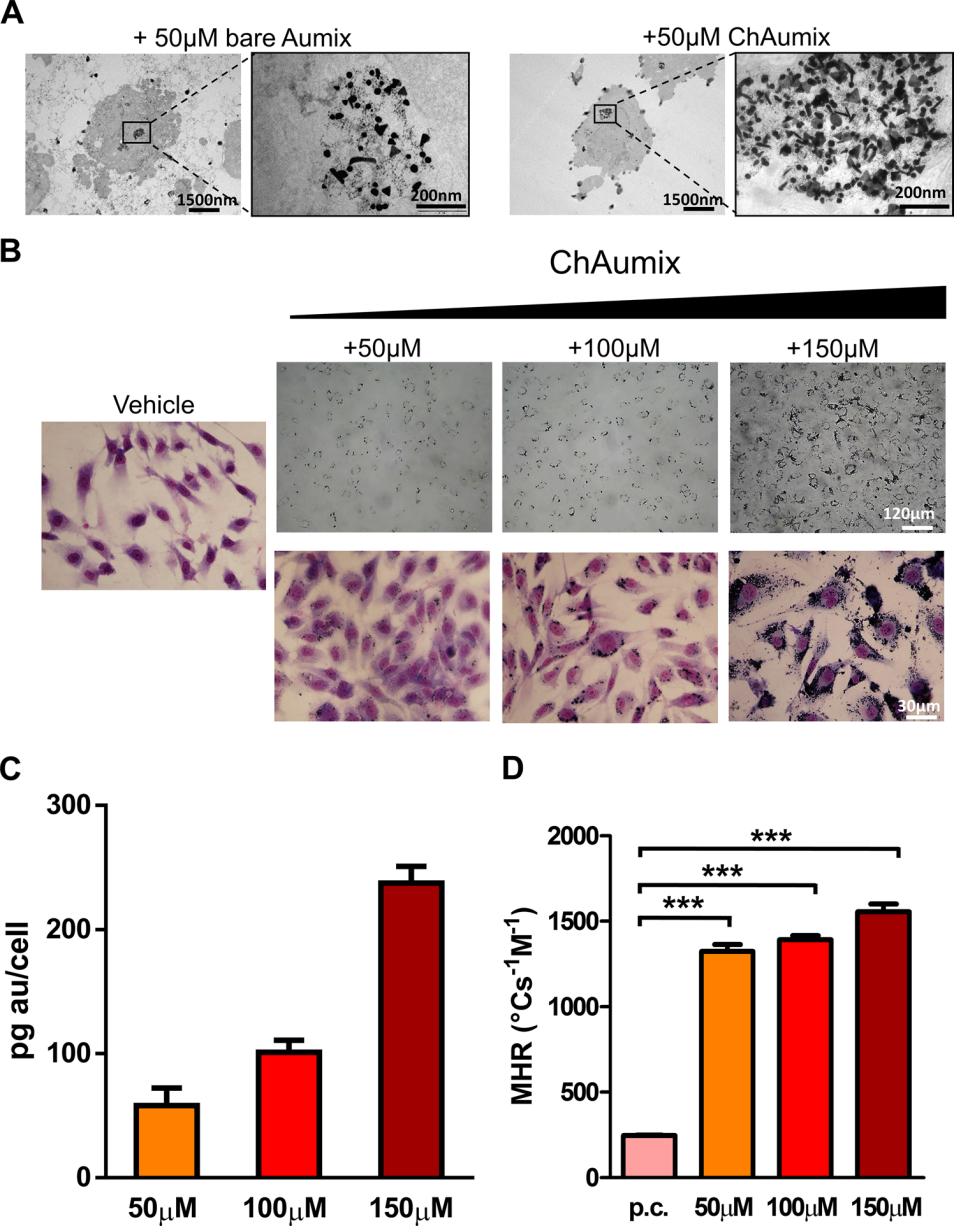
AuNP ECFC uptake (**A**) The uptake of uncapped (left side) and chitosan-capped (right side) Aumix by ECFCs. The nanoparticles are endocytosed and enclosed in phagosomes. A lower number of internal gold-rich phagosomes are observed for bare nanoparticles, with a lower packing density. (**B**) The effective endocytosis of ChAumix at increasing concentrations of Au in the incubation solution is easily observed also with an optical microscope on non-stained (upper images) or May-Grunwald Giemsa stained (lower images) samples. The inner gold content is represented by the black areas in the cells. (**C**) Dose-dependence of the cells enrichment resulting from ICP-AES measurements, (**D**) molar heating rates (MHR) of an as-made colloidal solution and Au-enriched ECFCs illuminated with the same light intensity (3 Wcm^−2^). For this test, 5 × 10^4^ ECFCs were used after the enrichment stage as described in the Experimental Section. P.c. refers to pure colloid (1.4 mM Au molar concentration).

To support the potential translational value of ECFCs as vehicles of ChAumix for thermoablation therapy we measured the ability to accumulate Au nanoparticles in ECFCs. Typical results of TEM analysis of the enrichment of ECFCs are illustrated in Figure 2A. First, the images show that, regardless of the different shapes or dimensions, all the populations of GNPs, are equally well internalized and mainly packed in phagosomes, without introducing cellular damage for both Aumix and ChAumix. Second, the density of packed nanoparticles is clearly higher in the case of ChAumix, and is consistent with a higher internalization likely due to their electrostatic attraction which enhances nanoparticle-cell membrane interaction. On the basis of these qualitative but nonetheless unambiguous findings, we selected ChAumix to load ECFCs. Exposure of ECFCs to increasing Au-mass concentration of 50, 100, 150 μM for 24 h, (from now on we refer to either ECFCs or A375 exposed to 50, 100, 150 μM Au as ECFCs (50, 100, 150) and A375 (50, 100, 150) resulted in a dose-dependent cellular uptake as observed even with a conventional optical microscope under white light illumination. Figure 2B clearly shows the internalized metal loads into ECFCs, identifiable as the black areas inside the cells. ICP-AES confirmed the qualitatively observed dose-dependent metal enrichment, with the results reported in Figure 2C. Remarkably high Au loadings have been found: 50 pg/cell, 100 pg/cell, 220 pg/cell for ECFCs(50), ECFCs(100) and ECFCs(150) respectively. Then, we irradiated Au-rich ECFCs to assess their photothermal behavior and evaluating their MHR, reported in Figure 2D together with the MHR of the as-prepared colloid. Noticeably, the MHR of the enriched cells, typically 1400°C s^−1^ M^−1^, is roughly 7-fold that of ChAumix in colloidal solution. This result is in agreement with the observation that closely packed nanoparticles behaves as improved heat generators [28], which can also explain the good heating performances found by Kang et alii [29], obtained by aggregating spherical nanoparticles via pH-induced modification of the nanoparticles surface electrical charge. In that case, from the data presented, a cellular MHR of 1190°C s^−1^ M^−1^ can be inferred, quite close to the MHR calculated for ECFCs.

**Figure 3:**
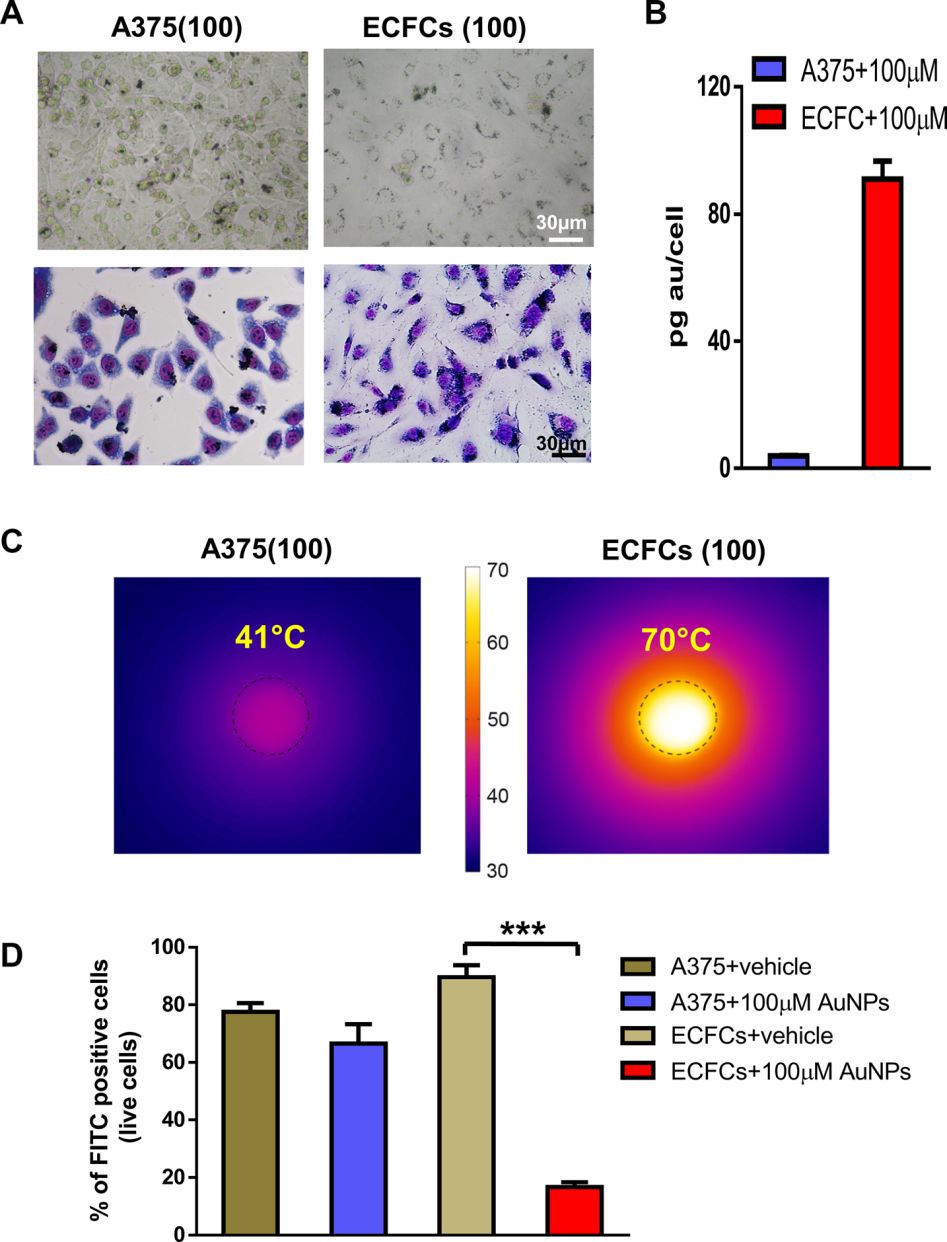
ECFC vs. A375: Au enrichment and photothermal properties (**A**) Images of A375 and ECFCs exposed to a 100 μM Au colloid and (**B**) the ICP-AES comparison of the respective Au-cargos. ECFCs are able to uptake an Au mass roughly 23-fold that one uptaken by A375 cells. (**C**) Thermographs of solutions of 1.5 × 10^5^Au-rich A375 cells (left) and an equal number of Au-rich ECFCs (right), irradiated with a 3 W cm^−2^ NIR laser (808 nm wavelength). In the former case, the maximum temperature (41°C) is reached after 6 minutes and doesn't exceed the hyperthermic value, while this is easily obtained for Au-rich ECFCs, that reach a temperature of 70°C after only 3 minutes of irradiation. (**D**) The viability of exposed ECFCs strongly reduced after the irradiation.

The advantage of the improved cellular uptake induced by the positively charged nanoparticles could also be in principle exploited to enrich the tumor mass with a direct inoculation of nanoparticles. For this reason, we tested *in vitro* the A375 capability to incorporate ChAumix and warm up upon NIR irradiation. The effects have been directly compared with those obtained with ECFCs(100), with the results shown in Figure 3. The difference in Au-load is already evident at the optical microscope analysis, (Figure 3A), but can be fully appraised by inspecting the ICP-AES results (Figure 3B), where ECFC(100) Au content, 100 pg/cell, comes out to be 23 times higher than 4.4 pg/cell of A375(100) cells. A similar enrichment, 7.9 pg/cell, was found for A375(150) cells. The marked difference in the gold uptake between A375 and ECFCs produces better thermal behavior in the latter case, as it will be shown in the subsequent NIR-irradiation tests performed on liquid PBS (Phosphate Buffered Saline) suspensions of Au-doped A375 and ECFCs (Figure 3C). The thermal kinetics of the two suspensions (not reported) allowed us to calculate their MHR, that resulted higher (4500°C s^−1^ M^−1^) for A375 than for ECFCs (1400°C s^−1^ M^−1^). However MHR does not take into account the loss of the large part of gold particles in the A375 uptake nor the effects of strong packing, as in the case of ECFCs. It is well known [28, 30] that strong nanoparticles packing reduces the interparticles distances producing a shift of the absorbance spectrum with a consequent loss of infrared light absorption per each particle and a reduction of the MHR. Luckily, the reduced MHR of ECFCs is widely balanced by the huge uptake of gold nanoparticles by the cells so that the heat power produced by enriched ECFCs is much higher, and, as a consequence, much higher temperatures can be reached in tumors.

Flow cytometry analysis of the fluorescence retained by viable cells confirmed that A375(100) and unloaded A375, exposed to the same NIR intensity, have comparable viability (Figure 3D). Conversely, the viability of ECFCs(100) showed a decrease close to 80% compared with ECFCs treated just with the vehicle (Figure 3D). These results indicate a clear correlation between the lower Au-uptake of A375 and the lower temperature raise while proving at the same time that Au-loaded ECFCs very efficiently absorb NIR light and warm up the surrounding medium well beyond the hyperthermic threshold.

### ECFCs phenotypical features vs. Au enrichment

**Figure 4:**
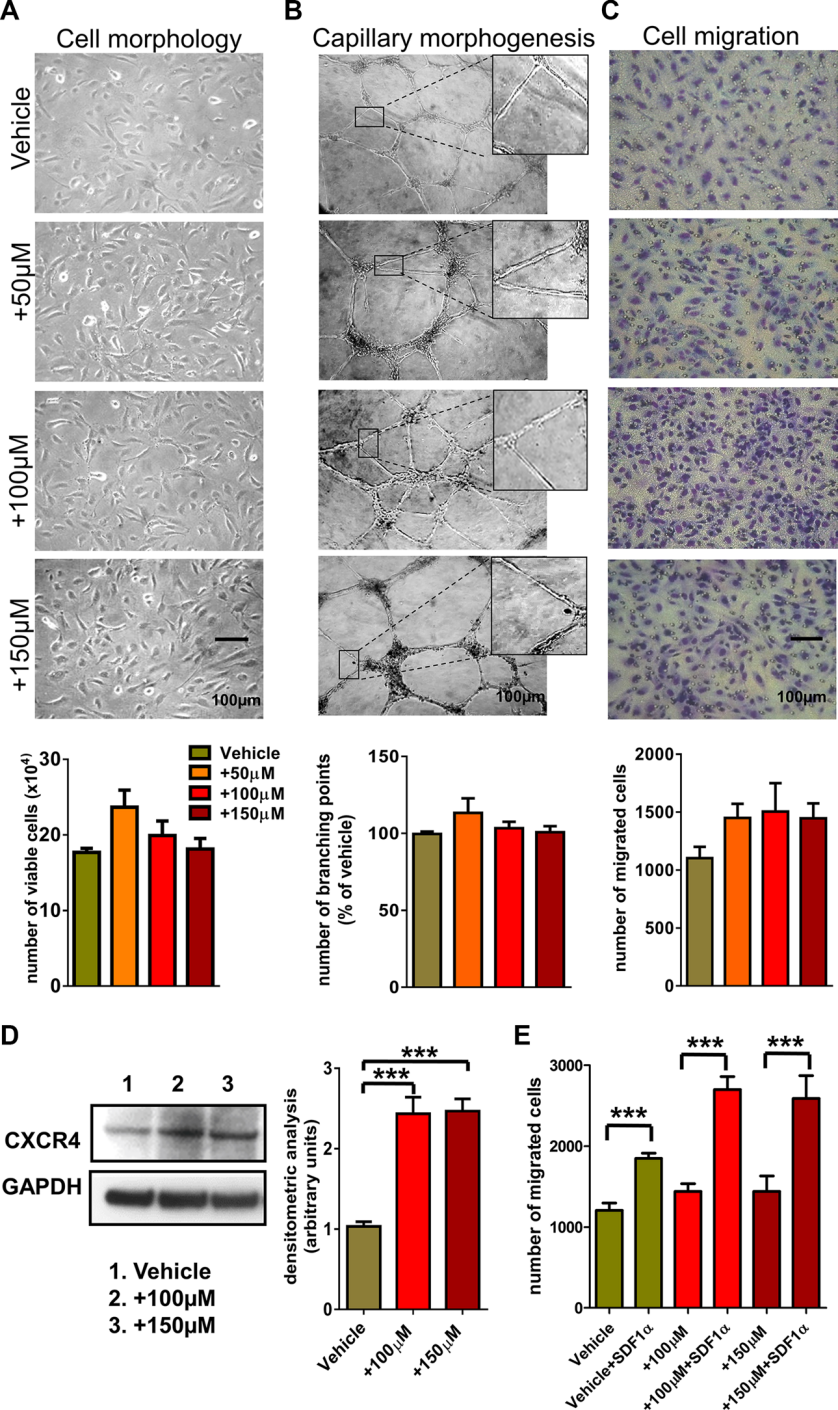
ECFC phenotypical features vs. Au enrichment (**A**) Cytotoxicity of gold nanoparticles after 24 h exposure on ECFCs: on the upper panel, morphological evaluation by bright field microscopy, on the bottom histogram representing viable cells (trypan blue-negative) counted with the aid of a Burker chamber, as described in Experimental Section. (**B**) ECFCs treated either with the vehicle or with increasing dose of ChAumix were cultured on Matrigel matrix supports. Cell organization was examined microscopically (a detailed enlarged structure is shown in the inset) taking random photomicrographs after 24h with the aid of a Nikon E 4500 photo camera (Nikon) on a Nikon TMS-F phase contrast microscope (Nikon Instruments) and quantified by manually counting the number of branching points/field reported as percent of control (ECFCs treated with the vehicle). Experiments have been performed three times in triplicate with analogous results. (**C**) Migration of ECFCs treated as in (B) was determined by Boyden chambers, as described in Experimental section. Data are expressed as means of migrated cells ± SD of three independent experiments performed in triplicate. (**D**) Western blotting detection of CXCR4 in ECFCs treated as in B) and GAPDH expression, as a control for protein loading. Histograms on the right report band densitometry, assuming 1 as the reference value of vehicle treated cells. (**E**) Migration of ECFCs treated as in (B) and in presence of 100 ng/ml of SDF1α, was determined by Boyden chambers, as described in Experimental section. Data areexpressed as means of migrated cells ± SD of three independent experiments performed in triplicate.

A sufficient intracellular ChAumix uptake, not compromising the viability and motility of ECFCs, is a critical issue. Hence we examined the incremental dose uptake profile along with cytotoxic and biological assay. ECFCs were incubated with increasing doses of ChAumix for 24 h and measurements of cell viability were conducted using WST1 (data not shown) or trypan blue assay as described in the Experimental Section. As shown in Figure 4A, ChAumix treatment does not affect cell morphology and does not reduce the number of viable cells. To further evaluate likely effects of GNPs on biological or functional cell activities, loaded and unloaded ECFCs were induced to capillary morphogenesis. As shown in Figure 4B ECFCs loaded with ChAumix were capable to form capillary network structures with the same effectiveness as untreated ECFCs and the degree of differentiation was then quantified as number of branching points. These results not only show that the Au-enrichment does not affect the ECFCs capability to differentiate in tubular-like structures, but also that gold nanoparticles are retained inside the cells after having been induced to differentiate. Since armed ECFCs will be used as cellular vehicles to deliver therapeutic GNPs to the tumor site, we further investigated whether nanoparticles uptake could affect cell migration. To this purpose ChAumix treated ECFCs were used for Boyden chamber assay. This Au-enrichment slightly increased the number of migrated cells compared to untreated ECFCs (Figure 4C). As we previously found that ECFCs home to tumor site by exploiting the CXCR4/SDF1 axis, we determined whether the presence of ChAumix was able to alter CXCR4 expression. ECFCs treated for 48 h with 100 μM and 150 μM Au-colloids showed significant increase of CXCR4 protein levels (Figure 4D). These results reveal the ability of ChAumix to modulate the expression of genes involved in cell motility such as CXCR4. Indeed, unexpectedly, we found a significant upregulation of CXCR4 expression, and a sensible increase of cell migration indicating an enhanced tumor tropism of Au enriched cells. To investigate the possible role of SDF-1/CXCR4 axis in ECFC migration, we performed a cell migration assay in presence of SDF1α ligand. In agreement with previously published evidence, the results showed that CXCR4, mediates the migration of these cells toward SDF1α, and notably Au loaded cells exhibited a greater chemotactic response to SDF1α compared with vehicle treated-ECFCs (Figure 4E). More importantly, the *in vitro* cell viability assay showed that the nanoparticles were harmless for ECFCs even at relatively high concentration suggesting that they might be appropriate photothermal agent in cancer therapy.

### *In vitro* killing of tumor cells by Au-rich ECFCs

**Figure 5:**
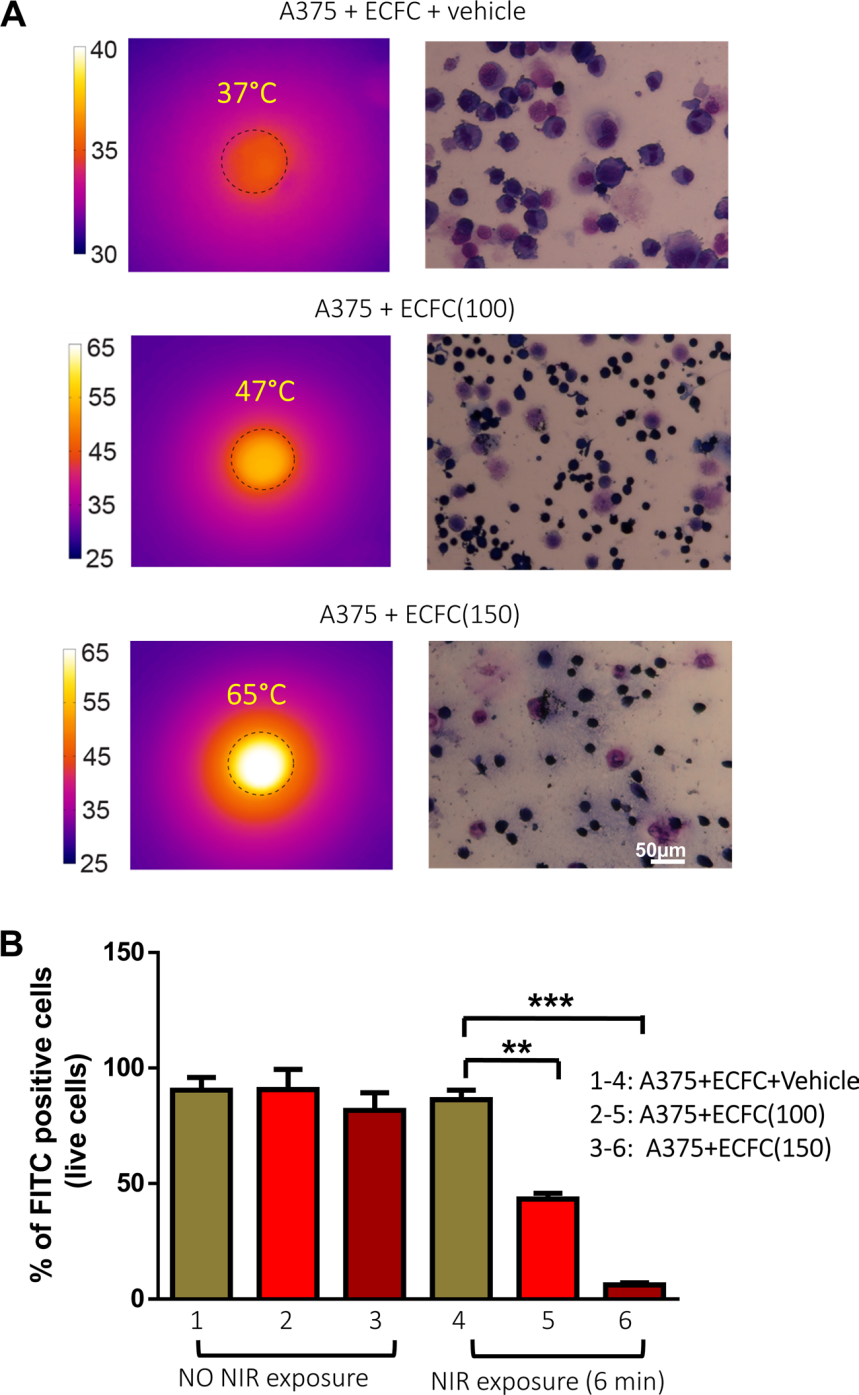
Photothermal effects of AuNPs on melanoma cells (**A**) (Left side) Thermographic images of the liquid mixtures of unloaded A375 and unloaded ECFCs or ECFCs(100,150), irradiated with a 808 nm wavelength laser source at the intensity of 4.8 W cm^−2^. While the temperature of the mixtures of unloaded cells doesn't raise significantly, hyperthermic regimes are reached when ECFCs (100,150) are present, reaching maximum temperatures of 47°C and 65°C, for the two enrichments respectively. Correspondent massive cells death is observed (right side) with microscope inspection and (**B**) confirmed by flow cytometry analysis of melanoma cells.

We assessed definitely the photothermal potentials of Au-enriched ECFCs by proving *in vitro* the effective thermolysis of undoped A375 cells by mixing them with ECFCs in a PBS environment with a 20:1 (A375 : ECFCs) ratio. 1 × 10^6^ CFSE stained A375 cells were mixed with 5 × 10^4^: a) vehicle-treated ECFCs, b) ECFCs (100), c) ECFCs (150), then the mixtures were irradiated with NIR laser light (808 nm wavelength, intensity: 3 Wcm^−2^). Figure 5A shows the thermographic images of cell suspensions after 6 minutes of irradiation. The temperature profiles for differently doped ECFCs show that hyperthermic regimes are reached for both mixtures, with maximum temperatures of 47°C and 65°C for ECFCs(100) and ECFCs(150) respectively, while the control mixture (A375+vehicle-treated ECFCs) didn't exceeded 37°C temperature. The flow cytometry analysis shows that NIR light irradiation of the A375 : ECFCs mixture caused a massive loss of A375 viability. In particular, 50% of melanoma cells were killed in presence of ECFCs(100), while almost a complete A375 loss was achieved with ECFCs(150) (Figure 5B). Morphologic examination after May-Grunwald staining confirmed the massive loss of viable cells, characterized by membrane disruption and cellular swelling induced by NIR exposure (Figure 5A).

These tests definitely show *in vitro* that heavily Au-doped ECFCs are able to warm up very efficiently tumor environment, and to kill unloaded cancer cells via hyperthermic heating.

### *In vivo* photothermal ablation of tumor tissue with Au-rich ECFCs

**Figure 6:**
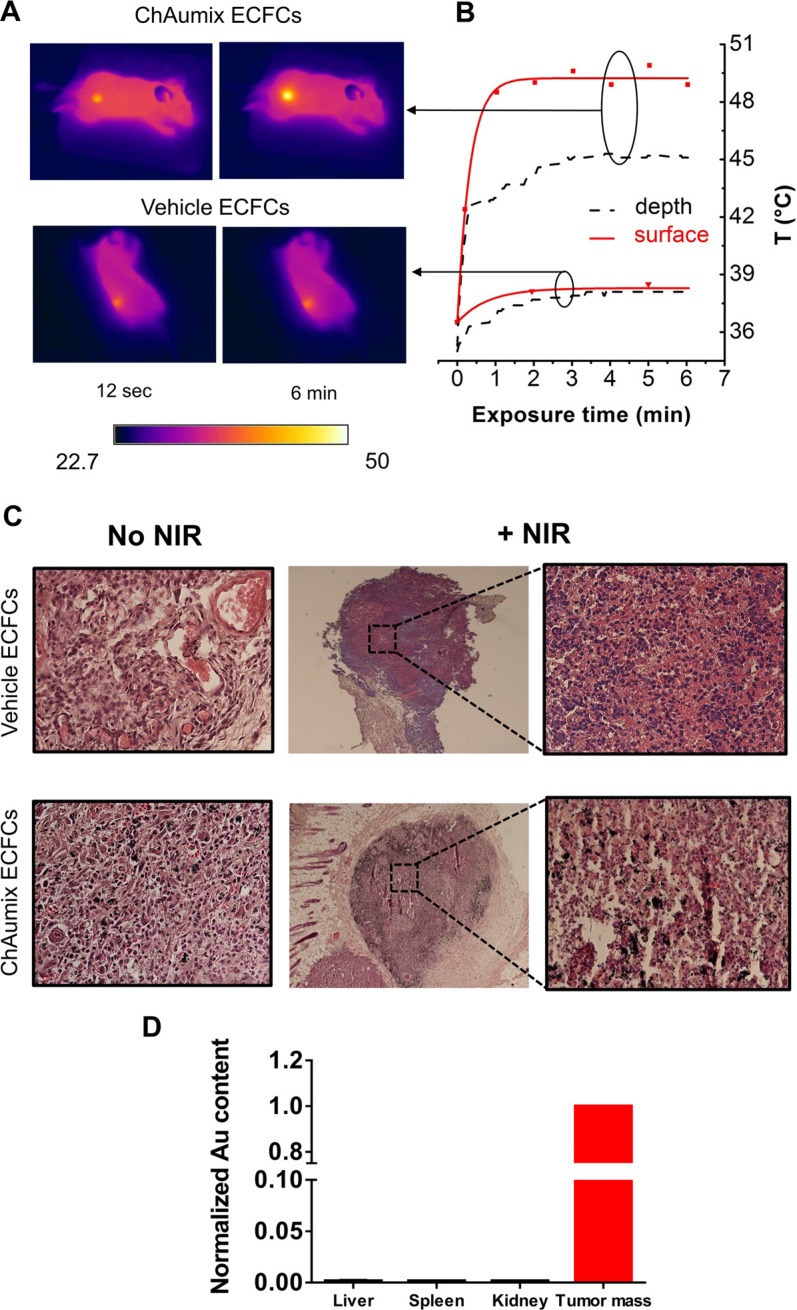
Photothermal ablation of human melanoma xenograft with Au-rich ECFCs (**A**) Thermographic images of the irradiated tumors treated with ChAumix-enriched EFCFs (upper part) or vehicle (lower part) and (**B**) the corresponding temperature growth measured with the FLIR thermocamera (red points) and with a temperature gauge (dashed black lines), placed at approximately 6 mm below the skin surface. The continuous red lines represent the exponential best fits of the experimental data. The initial growth rate of the upper curve, used to evaluate the MHR of the tumor-hosted Au-enriched ECFCs (see text), is 0.54°C s^−1^. Upper traces: temperature increase of the tumor treated with ChAumix-enriched ECFCs and with vehicle ECFCs (lower traces). (**C**) Histological Assessments of tumor tissues before and after NIR irradiation (1 W cm^−2^). (Pink: cytoplasm stained with eosin, Dark blue: nuclei stained with hematoxylin) (**D**) Gold amount in liver, spleen and kidney normalized to that one found in tumor mass.

Taking advantage on *in vitro* tests, we performed preliminary measurements to evaluate the *in vivo* photothermal potentials of Au-laden ECFCs. A mixture of 1.5 × 10^6^ viable A375 cells were injected into 8 weeks old CD-1 nude (nu/ mice) together with 3 × 10^5^ Au-rich ECFCs (100 pg/cell) or vehicle treated ECFCs. After the tumors reached a volume of 100 mm^3^, they were exposed to an 808 nm laser at a power density of 1 Wcm^−2^. The surface temperature was monitored during irradiation via a FLIR thermocamera, while the temperature monitoring at a depth of ~ 6 mm is performed with a thermocouple gauge. Figure 6A shows the thermographic images of tumor mass after 12 seconds and 6 minutes of irradiation. The temperature kinetics for the tumor with doped ECFCs show that hyperthermic temperatures are obtained both at the surface (50°C) and in depth (45°C) respectively, while in presence of unloaded ECFCs tumors didn't exceeded 39°C temperature (Figure 6B). Mice were sacrificed one week after the last NIR dose exposure and histological examinations of tumor tissues from group of mice injected with Au-enriched ECFCs confirmed the successful destruction of tumor cells due to the photothermal effect (Figure 6C). According to hematoxylin and eosin (H&E) staining results, common features of thermonecrosis such as loss of nucleus, cell shrinkages, and coagulation were found in tumor tissues from mice with Au-doped ECFCs and exposed to NIR, while viable cells with no obvious thermal damage in vehicle plus NIR treated group were comparable to No NIR group.

The gold amount found in tumor and RES organs such as spleen, kidneys and liver was measured with ICP-AES (Figure 6D). Noticeably, the gold amount in the analyzed RES organs is negligible and this means that, during the tumor growth, no significant gold loss occurred. Although other experiments are necessary to understand these findings, they seem to indicate, as previously reported [22], that the interaction of ECFCs through CXCR4 with SDF1 of tumor cells is strong enough. At the same time, as gold can in principle be exocytosed and drained out of the tumor, the above findings point to exclude this occurrence, in agreement with the resistance to exocytosis of chitosan-capped nanoparticles just previously observed for chitosan-capped iron-oxide nanoparticles [31].

From the best fitting of the temperature increase of the gold-enriched tumor mass (Figure 6B), a heating rate of 0.54°C s^−1^ is calculated. Considering the molar concentration of the Au mass in the tumor volume, a MHR of 252°C s^−1^M^−1^ is derived. This value might be compared with the one inferred from data of Li et alii [32], that injected in the tumor a gold mass of 105 μg. A heating rate of 0.16°C s^−1^ can be inferred from the presented data, corresponding to a MHR of 64°C s^−1^ M^−1^, that is 3.8 times lower than that found for Au-laden ECFCs.

## DISCUSSION

Anticancer drugs encounter several challenges to their therapeutic efficacy, including short half-life, drug resistance, and nonspecific distribution with potential side effects to healthy organs. Nanotechnology provides a powerful tool to circumvent these hurdles and result in improved efficacy. Even though during the last decade multifunctional nanoparticles (NPs) have been developed and modified to elongate circulation time and improve the accumulation of therapeutic drugs in tumor tissues, the massive uptake of NPs by tumor tissues is still a significant challenge. More than 90% of the injected NPs, *in vivo*, end up in or near normal tissues and organs such as liver, kidneys and spleen. Use of cells with tumor-homing capacity for delivery of nanomedicines is a relatively new and appealing approach in cancer therapy. In a recent study, Kang and coworkers [29] demonstrated that harnessing of MSCs as delivery vehicles of gold nanospheres led to 4-fold increase in tumor accumulation compared with intravenous injection of free nanoparticles. Although these results are promising, the biodistribution study still showed a low efficacy of tumor targeting after intravenous administration and hence the need to reduce their off-target accumulation and improve tumor tropic features. Moreover, though MSC phenotype can be accurately controlled within *in vitro* settings, it is challenging to control cell properties after transplantation because MSCs are at the mercy of the biological milieu. For instance, MSC immunosuppressive properties observed *in vitro* often do not correlate with *in vivo* function [33, 34].

In this work we show that Au-enriched ECFCs are suitable for human melanoma cellular photothermal therapy. ECFCs have the capacity to home, invade, migrate within, and incorporate into tumor structures. Moreover, ubiquitously available autologous blood is conveniently procured, can be easily expanded and does not induce immunological intolerance problems. For these reasons, ECFCs have been recently elicited as ideal candidates for tumor cell therapy [21, 22]. We have proved that they can be massively loaded with NIR-absorbing chitosan-capped nanoparticles without losing their phenotypical features. Rather, quite unexpectedly, we found a significant increase of CXCR4 expression along with a greater migration of Au enriched cells toward SDF1α. Moreover, the nanoparticles mix that we used has been prepared with a simple one-step protocol without the use of capping agents or pre-formed seeds and then stabilized with chitosan, a highly biocompatible and cheap polysaccharide widely used, for instance, in food industry. In spite of the inherent lack of homogeneity in the shapes/dimensions of the produced GNPs, these colloids contain a considerable number of NIR-absorbing nanoparticles that prove effective in heat production. Other kinds of popular NIR-tuned single-shaped GNPs, like Au-nanorods and Au-nanostars, show higher MHRs [27] about 7-fold higher as, for instance, in the favorable case of nanohexapods [32]. However, the better performances of purified ChAumix and of other NIR-tuned GNPs are paid with non-trivial issues in their preparation (i.e. multi-steps procedures, lossy purification steps, use of toxic surfactants). As a final remark, if from one side the presence of broadly dispersed ChAumix limits the absorption at a given excitation wavelength to a restricted number of tuned particles, on the other side the absorbance remains higher than the 90% of the maximum value in a wide band (~ 100 nm), and thus may comply more easily with laser users, hopefully in hospital. We measured Au loadings as high as 220 pg/cell, a cell enrichment that, at our knowledge, has not been reached so far [2, 29, 32]. This is due to the high efficiency of the enriching process, likely driven by the attractive forces between positively charged Au nanoparticles and negatively charged cells membranes in agreement with the observations reported by M. Pérez-Hernández [35]. Our findings show for the first time differential uptake between human melanoma and ECFC cells exposed to the same kind colloid at equal Au concentrations, confirming that, besides the complexity of the EPR effect and tumor heterogeneity, tumor cells have lower ability to accumulate gold in the cancer tissues compared to ECFCs and are thus more resistant to cell death induced by NIR exposure. Based on these results we can speculate that systemic administration of free ChAumix, *in vivo* will not be taken efficiently by tumor cells. Of note Au enrichment of A375 cells are of the same order of the highest ones reported for other kinds of tumor cells, that span in a wide range (from ~10^−3^ to ~10 pg/cell) [36–39] because of the multiplicity of factors that influence the GNPs uptake. It is worth noticing that the highest reported values of ~10 pg/cell [39] refer to Au-nanorods and chitosan-capped Au-nanostars, a circumstance that once more seems to confirm the increased GNP-cell membrane interaction due to the cationic decoration of nanoparticles.

After having been incorporated by ECFCs, chitosan-capped nanoparticles are closely packed in endosomes, so they can benefit of the efficient automatism that prevent their ejection from the cells via the lysosomal exocytic pathway, as clearly proved for chitosan-capped iron-oxide nanoparticles [31].

Both *in vitro* and *in vivo* tests have shown the excellent thermotransductive properties of enriched ECFCs and their ability to kill cancer cells at moderate NIR light intensities (*in vivo*: 1 Wcm^−2^). At our knowledge, we can favorably compare the heating performances of Au-rich ECFCs only with the data reported by Li et al. [32]; in such case, the MHR of Au-rich ECFCs is clearly higher. Based on these findings, doped ECFC might be triggered to induce death of tumor cells *in vivo*. The therapeutic impact of these results are relevant for the control of melanoma, which is one of the most aggressive skin cancer, notorious for its high multidrug resistance, easy to relapse and low survival rate.

As ECFCs have been found to avoid the accumulation in RES organs and specifically locate within tumors, the future opportunity of targeting enriched vectors via bloodstream seems a reasonable task. In this case, labeled cells with magnetic resonance imaging contrast agents, such as iron oxides super-paramagnetic nanoparticles (SPIONs) or gadolinium, can work as diagnostic probes used in nuclear medicine or optical imaging. Thus, a new “theranostic” strategy based on specific enriched ECFCs might help clinicians to contrast tumor progression and early metastases.

## MATERIALS AND METHODS

### Cell lines

Endothelial Colony-Forming-Cells (ECFCs), a subpopulation of EPCs, were isolated from > 50 ml human umbilical cord blood (UCB) of healthy newborns, as described in [40] after maternal informed consent and in compliance with Italian legislation, and analyzed for the expression of surface antigens (CD45, CD34, CD31, CD105, ULEX, vWF, KDR, uPAR) by flow-cytometry. The melanoma cell line A375 was obtained from American Type Culture Collection (Manassas, VA) and grown in Dulbecco's modified Eagle's medium (DMEM) with 10% FBS (Euroclone).

### Preparation and characterization of ChAumix

Gold nanoparticles were prepared by chemical reduction of HAuCl_4_, following the recipe of [24]. Basically, 3 mM Na_2_S_2_O_3_ solution was added to 1.7 mM HAuCl_4_ solution and vortexed for 20 seconds at room temperature. The molar ratio was adjusted in order to obtain a plasmonic band close to 800 nm. The solution was then left to react undisturbed and the extinction spectrum was checked via UV-Vis absorption spectroscopy. The colloidal solution was then stabilized by adding a 1% acetic acid solution of chitosan (Au/chitosan molar ratio = 5) and incubated overnight with gentle shaking. Apart from the mandatory sterilization procedure with standard autoclave treatments before biological tests, the colloid wasn't subjected to further handling. The final Au concentration in the colloidal solutions was 1.4 mM equivalent to a mass concentration of 280 μg/mL. Ultrapure Millipore water (resistivity 18 MΩ × cm) MilliQ water was used as solvent. The sample ChAumix solution, used to verify with UV-Vis spectroscopy the nanoparticles capping, was purified from chitosan excess by centrifugating 2 mL of the prepared solution at 10000 rpm for 5 minutes. The surnatant was then removed and the remaining colloid was resuspended in ultrapure water and used for spectroscopic tests. Tetrachloroauric(III) acid (HAuCl_4_) and thiosulphate (Na_2_S_2_O_3_) were purchased from Sigma Aldrich and used as received. High-molecular weight chitosan (~106 Da; 79% deacetylation degree) was obtained from Heppe Medical. All glassware was cleaned with piranha solution (H_2_SO_4_ : H_2_O_2_ 3:1 v/v).

The UV-Vis analysis of the fabricated colloids was performed by illuminating a 2 mL liquid sample (optical thickness: 2 mm) placed in a quartz cuvette with a couple of deuterium and halogen lamps (Avantes Avalight-DH-S-BAL), and by recording the absorbance spectra with an Avanspec-3648 spectrometer. The ζ-potential of the colloidal solutions was measured with a Malvern Zetasizer Nanoseries apparatus. The morphology and dimensions of the nanoparticles were checked with a Philips CM12 cryo-Gatan UHRST 500 @ 100 kV transmission electronic microscope (TEM). The sample was prepared by dropping the colloidal solutions onto carbon-coated Cu grids and letting them dry.

### TEM analysis of Au-enriched ECFCs

The ECFCs were seeded in 6-well plates at a density of 1.5 × 10^5^ cells per well and allowed to attain 70% confluence. The cells were then incubated with a culture medium (2 mL per well) containing diluted Au-colloidal solutions at a concentration of 50 μM. The cells were collected by trypsin treatment after 24 h of incubation, and centrifuged at 1000 rpm for 5 min in a 1.5 mL Eppendorf tube. The cell pellet was then fixed in isotonic 4% glutaraldehyde and 1% OsO_4_, dehydrated, and embedded in Epon epoxy resin (Fluka, Buchs, Switzerland) for electron microscopic studies. Ultrathin sections were stained with aqueous uranyl acetate and alkaline bismuth subnitrate, viewed and photographed under a JEM 1010 transmission electron microscope (Jeol, Tokyo, Japan) equipped with a MegaView III high-resolution digital camera and imaging software (Jeol).

### Inductively coupled plasma atomic emission spectroscopy (ICP-AES)

The ECFCs were seeded in 6-well plates at a density of 1.5 × 10^5^ cells per well and then incubated with a culture medium (2 mL per well) containing ChAumix at increasing concentrations 50, 100 and 150 μM for 24 h. The cells were then washed 2 times with phosphate buffered saline (PBS, Invitrogen), detached with a trypsin treatment and the cell number was counted using a hemocytometer. The cell pellets were collected by centrifugation, lyophilized, and placed in centrifuge tubes (one pellet per tube). Then 400 μL of aqua regia were added to each tube to completely dissolve the cells and their gold content. The amount of Au was measured by Elan DRC II ICP-MS (Perkin Elmer, Waltham, MA).

### Cell viability determination

The viability of ECFCs was determined by i) trypan blue staining and ii) cell proliferation assay using WST-1 reagent (Roche). Cells (1.5 × 10^5^) were seeded in 6-well plates and allowed to attach overnight. On the next day Au colloidal solutions were added at the indicated concentrations. 24 h later 20 μL of cells was aseptically transferred to a 1.5 mL clear Eppendorf tube and incubated for 3 min at room temperature with an equal volume of 0.4% (w/v) trypan blue solution prepared in 0.81% NaCl and 0.06% (w/v) dibasic potassium phosphate. Viable and nonviable cells (trypan blue positive) were counted separately using a dual-chamber hemocytometer and a light microscope. The means of three independent cell counts were pooled for analysis. WST-1 is a water-soluble sulfonated tetrazolium salt that is cleaved by cellular succinate-dehydrogenases in living cells, yielding dark blue formazan. Damaged or dead cells exhibit reduced or no dehydrogenase activity. Briefly, cells were seeded into the wells of a 96-well plate at a density of 5 × 10^4^ cells per well, incubated with AuNPs at various concentrations for 24 h. 10 μL of WST-1 in PBS was added to each well, and incubated at 37°C for 1 h. Absorbance at 450 nm (reference at 630 nm) was measured by a Multiskan JX microplate reader.

### Capillary morphogenesis

*In vitro* capillary morphogenesis was performed as described in [41] in tissue culture wells coated with Matrigel. Cultures were pre-treated with/without nanoparticles (50–150 μM) for 24 h and then aliquots of ECFCs (1.8 × 10^4^) were plated in EBM-2 medium, supplemented with 2% FCS and incubated at 37°C-5% CO_2_. Morphogenesis was evaluated taking pictures at different times with the aid of a Nikon E 4500 photocamera (Nikon) on a Nikon TMS-F phase-contrast microscope (Nikon Instruments). Six to nine photographic fields from three plates were scanned for each point. Results were quantified by manually counting the number of networks branching out from a branch point/node per field expressed as percentage in respect to control set as 100%.

### Invasion assay

The Boyden chambers were used to evaluate spontaneous and stimulated invasion (chemoinvasion) of cells through Matrigel-coated 13mm diameter polycarbonate filters with 8 μm–pore size. Matrigel (BD, Biosciences) was diluted to the desired final concentration (50 μg/filter) with cold distilled water, applied to the filters, dried under a hood, and reconstituted with serum-free medium. ECFCs were pre-treated with/without nanoparticles (50–150 μM) for 24 h and then aliquots of cells (2 × 10^4^) were resuspended in EBM-2 medium containing 2% of FBS and placed in the upper compartment of the chamber. For assessment of spontaneous invasion, same medium was added to the lower compartment. For assessment of chemoinvasion, 100 ng/ml of SDF1α was dissolved in EBM-2 containing 2% of FBS and placed in the lower wells. After 6 h incubation at 37°C in a humidified atmosphere containing 5% CO_2_, the filters were recovered, cells on the upper surface mechanically removed while cells adhered to the lower filter surface were fixed with absolute methanol and stained with Diff-Quick staining solution. The number of cells moving across the filter was used as the measure of mobilization. Cells were counted in a double-blind manner in five different microscopic fields for each condition with a light microscope. All experiments were performed three times in triplicate. Migration has been expressed as the mean ± SD of the number of total cells counted per well.

### Western blot analysis

Harvested cells were resuspended in 20 mM RIPA buffer (pH 7.4) containing a cocktail of proteinase inhibitors (Calbiochem, Merck, Darmstadt, Germany) and treated by sonication (Microson XL-2000, Minisonix, Farmingdale, NY, USA). Proteins were assayed by the BCA Protein Assay (Thermo Scientific, Rockford, IL, USA), analyzed by SDS-PAGE and western blotting. Membranes were probed with primary antibodies against: CXCR4 (Santa Cruz Biotechnology, Santa Cruz, CA, USA) anti-GAPDH (mAbcam 9484) as loading control. Suitable peroxidase-conjugated IgG preparations (Sigma-Aldrich) have been used as secondary antibodies; the ECL procedure was employed for development.

### Photothermal properties of the ChAumix colloid

150 μL of the produced colloid (optical density = 0.7) were placed into one well of a 96-wells plate, and were irradiated with a CW-operating laser diode emitting at 808 nm wavelength with three different light intensities, namely 0.5, 1, 2 W cm^−2^. Laser light was delivered through a multimode optical fiber and collimated in a spot size of 5 mm diameter. The temperature raise was monitored during the irradiation with a thermocamera FLIR B335 (Spectral range 7.5–13 μm, sensitivity: 50 mK) equipped with a 18 mm focal length objective and a field of view (FOV) of 25° × 19° and recorded each 2 minutes. The images were elaborated with InfraRecorder software (version 0.52.0.0).

### *In vitro* photothermal treatment of Au-enriched cells and A375:ECFCs stoves mixture

To evaluate cell damage induced by NIR exposure, cells were labelled with carboxyfluorescein diacetate succinimidyl ester dye (Cell Trace CFSE; Molecular Probe, LifeTtechnology) which easily cross the plasma membrane and covalently binds to amine groups in proteins, resulting in long-term dye retention within the cell.

In a first test, we compared the photothermal behavior of Au-loaded A375/ECFCs. Cells were treated for 24 h with 100 μM ChAumix. After 24 h incubation, the growth medium was removed and the cells were washed several times with PBS to remove the free ChAumix that were not taken up by the cells. The cells were subsequently subjected to trypsin treatment and labeled with CFSE to evaluate the effect of cell loss after NIR exposure. 150 μl of cell suspension composed by 1.5 × 10^5^ of CFSE stained A375 treated with vehicle or with ChAumix or by 1.5 × 10^5^ of CFSE-stained ECFCs treated with vehicle or ChAumix were exposed to a CW NIR light laser at 808 nm (4.8 W cm^−2^) for 6 minutes. After laser exposure, the fluorescence retained by viable cells was measured by flow cytometry. In the cocolture experiment 150 μl of cell suspension composed by 1.0 × 10^6^ ofCFSE stained A375 were mixed either with 5 × 10^4^ vehicle-treated ECFC or with AuNPs loaded ECFC and were exposed to a continuous red light laser at 808 nm (4.8 W cm^−2^) for 6 minutes. After laser exposure, the fluorescence retained by A375 viable cells was measured by flow cytometry. Morphological changes were microscopically examined either by phase contrast or methanol-fixed cells stained with May-Grunwald using a Digital Camera System Leica DC 200 (Leica Microsystems, Inc. Bannockburn, IL, USA).

### *In vivo* photothermal treatment of tumors

All *in vivo* procedures were approved by the ethical committee of Animal Welfare Office of Italian Work Ministry and conformed to the legal mandates and Italian guidelines for the care and maintenance of laboratory animals. Six- to eight-week-old female athymic nude mice were purchased from Charles River. Evaluation of the therapeutic local effect of Au-laden ECFCFs on the tumor onset, were performed by co-injecting 1.5 × 10^6^ A375 cells in the flank of nude mice together with 0.3 × 10^6^ vehicle treated ECFCs (4 mice) or Au-laden ECFC (6 mice). In order to determine tumor volume, the greatest longitudinal diameter (length) and the greatest transverse diameter (width) were determined with external caliper. Tumor volume based on caliper measurements were calculated by the following formula; tumor volume = length × width^2^ × 0.5. Tumors reaching approximately 100 mm^3^ in volume were selected for the study. During the procedure, mice were anesthetized with isoflurane. The vehicle-treated mice group and the AU-laden ECFCs were exposed to NIR light three times each other day. During heating, thermal images were collected for mice before heating, and at 12 seconds, 1, 2, 3, 4, 5 and 6 minutes into the heating process. Throughout heating, the temperature of the tumors was monitored by thermocamera FLIR B335 as previously described. To prove that the effect on tumor was due to hyperthermia one group of tumor bearing mice inoculated with Au-doped ECFCs were not exposed to NIR irradiation. The animals were monitored daily and were sacrificed one week after the last NIR treatment. The histological analysis of tumor mass was performed as described below. Removed tumors and RES organs were fixed overnight at 4°C in formalin (5% in PBS) for histological analysis performed on paraffin-embedded sections (6 μm). The sections of tumor tissue was stained using hematoxylin and eosin and examined using an optical microscope.

To quantify the amount of gold in the tumor and organs, resected mouse tissues were prepared and analyzed using ICP-MS. Samples were frozen in liquid nitrogen and weighed, then digested in aqua regia and prepared as previously described.

### Statistical analysis

Statistical analysis was performed using 1 way ANOVA, Bonferroni post hoc test (**P < 0.01, ***P < 0.001). Independent experiments were performed at least 3 times in triplicate. Graphical representations were performed using GraphPad Prism version 5 (GraphPad Software, San Diego, CA).
